# Plectin as a prognostic marker in non-metastatic oral squamous cell carcinoma

**DOI:** 10.1186/s12903-015-0084-9

**Published:** 2015-08-26

**Authors:** Oddveig G. Rikardsen, Synnøve N. Magnussen, Gunbjørg Svineng, Elin Hadler-Olsen, Lars Uhlin-Hansen, Sonja E. Steigen

**Affiliations:** Department of Otorhinolaryngology, University Hospital of North Norway, Tromsø, Norway; Department of Medical Biology - Tumor Biology Research Group, Faculty of Health Sciences, UiT The Arctic University of Norway, Tromsø, Norway; Department of Clinical Pathology, University Hospital of North Norway, N-9038 Tromsø, Norway

## Abstract

**Background:**

Oral squamous cell carcinoma (OSCC) is associated with a poor 5-year survival rate. In general, patients diagnosed with small tumors have a fairly good prognosis, but some small tumors have an aggressive behavior leading to early death. There are at present no reliable prognostic biomarkers for oral cancers. Thus, to optimize treatment for the individual patient, there is a need for biomarkers that can predict tumor behavior.

**Method:**

In the present study the potential prognostic value of plectin was evaluated by a tissue microarray (TMA) based immunohistochemical analysis of primary tumor tissue obtained from a North Norwegian cohort of 115 patients diagnosed with OSCC. The expression of plectin was compared with clinicopathological variables and 5 year survival.

**Results:**

The statistical analysis revealed that low expression of plectin in the tumor cells predicted a favorable outcome for patients with non-metastatic disease (*p* = 0.008). Furthermore, the expression of plectin was found to correlate (*p* = 0.01) with the expression of uPAR, which we have previously found to be a potential prognostic marker for T1N0 tumors.

**Conclusions:**

Our results indicate that low expression of plectin predicts a favorable outcome for patients with non-metastatic OSCC and the expression level of plectin may therefore be used in the treatment stratification for patients with early stage disease.

## Background

Squamous cell carcinoma (SCC) accounts for more than 90 % of the malignant neoplasms of the head and neck region [1, 2]. Head and neck SCC (HNSCC) have a 5-year survival rate of approximately 50 %, [3, 4] and are ranked as the eighth leading cause of cancer death worldwide [5].

OSCC includes tumors located in the mobile tongue, floor of the mouth, buccal mucosa, gingival rim and the hard and soft palate. The most widely used classification-system of OSCC is the TNM-system by the Union for International Cancer Control [6]. This system is used to describe the anatomical extent of the disease at diagnosis based on primary tumor size and extent of tumor growth (T), regional lymph node metastasis (N) and distant metastasis (M), and is the basis for stage grouping these patients [7, 8]. By morphological assessment, tumors are graded as well, moderate and poorly differentiated [1]. The TNM-classification, and to a lesser extent, the grade of differentiation, are often used as predictors for outcome. However, OSCCs are molecularly heterogeneous and tumors with identical TNM-classification and differentiation grade may differ in aggressiveness and response to treatment [7, 9]. This unpredictable behavior raises the need for a diagnostic tool that can provide more reliable prognostic information.

A risk model based on histological analysis of the tumors has been proposed [10–12]. However, in a recent study this model did not predict the outcome of patients with tongue SCC [13]. Several previous reports have proposed different biomarkers, such as p53, EGFR, Ki67 and E-cadherin as prognostic markers for OSCC, [14–16] though none have been implemented in clinical practice. In an attempt to find new and better prognostic markers we have in this study focused on plectin Plectin is a large intracellular cytoskeletal linker protein (>500-kDa) that has been found to be important in cytoskeletal network organization. It is expressed in normal skin and in the epithelial lining of the gastrointestinal-tract, muscle cells and endothelial cells of vessels, [17] as well as in cancer arising in the esophagus, stomach, lung, pancreas and the oral cavity [17, 18]. Plectin is localized at the inner side of the plasma membrane where it is associated with intermediate filaments (IF), microtubules and microfilaments [19]. In hemidesmosomes, plectin interacts with the cytoplasmatic tail of the integrin β4 subunit. Defects in the plectin gene have been found in the severe, hereditary, skin blistering hereditary disease epidermolysis bullosa simplex, emphasizing the importance of the protein in normal epithelial cells [20]. Plectin affects mechanical, as well as dynamic properties of the cytoskeleton, and (at least in keratinocytes) plectin-deficiency has been shown to result in increased migration rates probably through activation of the Erk1/2 pathway [21]. In a study investigating HNSCC, Katada et al. found that the survival rate of patients with high plectin expression in their cancer cells was significantly decreased, and the frequency of recurrences significantly increased, compared to patients with low plectin expression [22].

In a recent study we showed that the urokinase plasminogen activator receptor (uPAR) and plasminogen activator inhibitor-1 (PAI-1) may be prognostic markers in early stage OSCC [23]. uPAR is a constituent of the plasminogen activator (PA) system, and both urokinase plasminogen activator (uPA) and uPAR are linked to increased proteolytic activity and migration of cancer cells. uPA converts plasminogen into the active serine protease plasmin, a broad spectrum protease that can degrade many different types of extracellular matrix proteins in addition to activate latent growth factors and matrix metalloproteases [24, 25]. By binding of uPA to uPAR, cancer cells can direct the proteolytic activity to the cell surface [26]. As with plectin, the increased expression of uPAR has been linked to the phenomenon epithelial-mesenchymal transition (EMT) [27–29].

In this TMA-based study, low expression of plectin was found to be a marker for a favorable prognosis in non-metastatic OSCC. Plectin and uPAR showed similar staining patterns in the tumors and there was a significant correlation between plectin and uPAR expression.

## Methods

### Specimens

This was a retrospective study using formalin fixed, paraffin embedded tumor samples from 115 patients with histologically verified primary OSCC in the period 1986–2002 collected from the archives of the Department of Clinical Pathology, University Hospital of North Norway. To secure a homogenous cohort, tumors with verrucous growth patterns as well as tumors from patients with previous cancer, or prior radiotherapy to the head and neck area were excluded. All patients presented with primary disease located in the oral cavity, including mobile tongue, floor of the mouth, buccal mucosa, gingiva and hard and soft palate. Relevant clinical data and TNM-classification were retrieved from patients’ files, including pathology reports, Statistics of Norway and the Cause of Death Registry. The N and M statuses were determined by clinical and radiological examination. The normal tongue mucosa tissue was obtained from tissue adjacent to the tumor tissue. The study was approved by the Regional Committees for Medical and Health Research Ethics, Northern Norway (No. 22/2007). The patient information was de-identified prior to analysis. The ethics committee deemed it unnecessary to obtain written or oral consent from the participating patients.

### Tissue microarray (TMA)

A morphologically representative region of each tumor was identified on a hematoxylin and eosin (HE)-stained slide, and cores for the TMA were harvested from the corresponding tissue block using a Beecher Instruments Micro Tissue Arrayer. Eight cores of 0.6 mm were taken from the selected regions of the donor block of each tumor and inserted into recipient paraffin microarray blocks. Four μm thick sections of the fixed, paraffin embedded TMA tissue were cut with a microtome and placed on Superfrost slides. HE-staining and immunohistochemical cytokeratin-staining was performed to verify the presence of tumor tissue.

We experienced a loss of 16.5 % of the cores during preparation, and of the preserved cores, 8.4 % contained too few tumor cells to be evaluated. This loss due to technical issues is moderate compared to other reports [30, 31]. From each patient a mean number of 3.82 cores stained and evaluated for plectin expression.

Evaluated from each patient was 3.82.

### Immunohistochemistry (IHC)

All slides were deparaffinised and rehydrated. The plectin antigen retrieval was enhanced by boiling in a pressurized system with 10 mM citrate buffer (pH 7.0). Further, the slides were incubated 30 min with 3 % H_2_O_2_ to block endogenous peroxidase activity, and incubated one hour with 10 % goat serum (Dako, Glostrup, Denmark) in phosphate buffered saline (PBS) (Dako, Glostrup, Denmark) to reduce unspecific staining. Rabbit primary monoclonal antibody against plectin (ab32528, Abcam, Cambridge, MA) was diluted 1:200 in Dako antibody diluent (S3022, Dako, Glostrup, Denmark), and incubated overnight at 4 °C. Subsequently, bound antibodies were visualized using the anti-rabbit Envision Plus System (K4011, Dako, Glostrup, Denmark). The slides were washed in PBS ^w^/0.1 % Tween-20 with high concentrations of salt (0.4 M NaCl) and pH 6.0 (PBSTS6) after incubation with primary and secondary antibodies.

The anti-plectin antibody (ab32528) has previously been described and validated as a good marker for pancreatic adenocarcinoma [18]. As a negative control, pancreatic carcinoma tissue was treated according to the same staining protocol, but the primary antibody was omitted. No staining was seen indicating that the secondary antibody gave no unspecific staining in the tissue (data not shown).

Staining of normal oral mucosa showed specific and strong staining of muscle tissue in blood vessels, serving as positive control.

The uPAR staining was performed according to previous describes protocols [23].

### Immunohistochemical scoring

All cores were examined by an experienced pathologist (SES) and a trained head and neck surgeon (OR) without knowledge of clinical outcome. The scoring was semi-quantitative, [32, 33] and the staining index (SI) calculated as a product of staining intensity (none (0), weak (1), moderate (2) or strong (3)) and proportion of positive tumor cells (no staining (0), <10 % (1), 10–50 % (2), 51–80 % (3) or >80 % (4)). Thus, the SI for each core differed from a minimum value of zero to a maximum of 12, and the patient’s final score was the mean SI of all cores evaluated. Tumors with score above the median value were classified as high expressers’, while those with score under median value were classified as having a low expression of the biomarker.

### Immunofluorescence (IF)

For IF analysis the slides were pretreated as described in the IHC protocol for plectin. A rabbit polyclonal anti-plectin antibody (ab83497, Abcam, Cambridge, MA) recommended for IF was used. The antibody was diluted 1:10 in Dako antibody diluent (S3022, Dako, Glostrup, Denmark) and incubated for 3 h at room temperature. After the slides had been washed with PBSTS6, they were incubated overnight at 4 °C with the mouse monoclonal anti-human uPAR antibody (#3936, Sekisui Diagnostica, Stamford, CT, USA) at a 1:10 dilution in a buffer of 10 % goat serum (Dako North America, Carpintera, CA, USA) in PBS with 1 % BSA and 0.3 % Tween-20, pH 6.0. After incubation the slides were washed with PBSTS6. The secondary antibodies (Alexa Fluor 488, goat-anti rabbit, A11034 and Alexa Fluor 647, goat-anti mouse, A21236, Invitrogen, Carlsbad, CA) were mixed and diluted 1:200 in PBS. The slides were mounted and counterstained with DAPI antifade (DAPI in Fluorgard, 0.5 g/ml, Insitus, Albuquerque, NM) and sealed with nail polish. The slides were observed and photographed using Leica TCS SP5 confocal laser microscope with Leica Application Suite Advanced Fluorescence software (Leica Microsystems AG, Wetzlar, Germany).

The specificity of the polyclonal anti-plectin antibody (ab83497) used in the immunofluorescence staining was tested by staining pancreatic adenocarcinoma. Similar to the monoclonal anti-plectin antibody, the polyclonal anti-plectin antibody specifically stained pancreatic cancer cells. Furthermore, Western blotting of whole muscle cell lysate showed that the antibody gave a band of the approximately 500 kDa corresponding to the size of plectin (data not shown).

### Statistics

All data were tabulated and analyzed using the IMB SPSS Statistics (Chicago, IL), version 21. Associations between different categorical variables were assessed by the Pearson`s chi-square test. Univariate analyses of the different variables’ influence on time to disease specific survival were performed using the Kaplan-Meier method, and statistical significance between categories was estimated by the log-rank test. Statistically significant values from COX univariate analyses were entered into a multivariate analysis using the backward stepwise Cox regression model. Disease specific death (DSD) was defined as patients dying form OSCC and not from unrelated conditions. These data were obtained from the “Cause of Death registry in Norway. Correlation between bivariates was calculated with Spearman’s rho. The cut-off for low and high expression for each biomarker was set at the median value of the patient’s final score, and was 5.60 for plectin and 5.63 for uPAR. All results were considered significant if *p* < 0.05, and reported according to the REMARK guidelines by McShane et al. [34] The starting point was defined as time of diagnosis, and the last day of follow up was January 1^st^, 2012.

## Results

In the present study the prognostic value of plectin in OSCC was evaluated by a TMA-based immunohistochemical analysis of primary tumor tissue obtained from a North Norwegian cohort of 115 patients. The cohort consisted of 64 men and 51 women, with a mean age of 65.2 years (men 64.4/women 66.0) at diagnosis [23]. The majority of the cases presented with small tumors (T1, 34 % or T2, 37 %), no lymph node metastasis (N0, 63 %) and a well or moderately differentiated tumor (90 %). As expected, tumor size (T) and the presence of lymph node metastasis (N+) correlated significantly with disease specific death (DSD), while the grade of differentiation did not.

### Immunohistological staining pattern

**Fig. 1:**
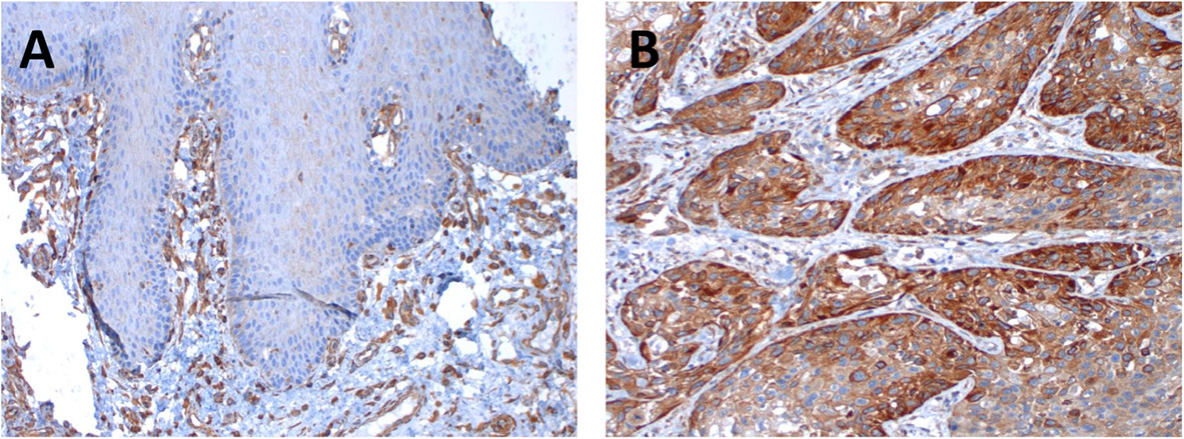
Immunohistochemical staining for plectin of normal tongue mucosa (**a**) and oral squamous cell carcinoma (**b**). The normal mucosa showed only faint staining while the blood vessels in the stroma were strongly stained. The staining of plectin positive tumor cells was strong, and the stroma showed almost no staining

**Fig. 2:**
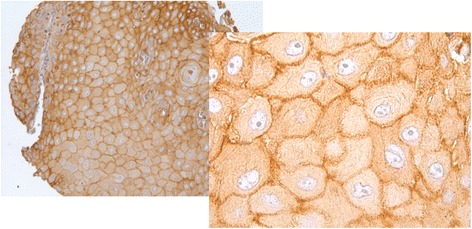
Immunhistochemical staining pattern in TMA cores of oral squamous cell carcinoma. Plectin staining was mainly at the plasma membrane, but also some cytoplasmic staining was seen

Plectin scarcely stained normal tongue mucosa that served as control material, while the blood vessels in the underlying stroma displayed stronger staining (Fig. 1a). In plectin-positive OSSC tumors the staining was relatively homogeneous throughout the tumor (Fig. 1b). The cancer cells were highly positive while the adjacent extracellular matrix showed less reactivity. The staining of the cancer cells was mainly confined to the plasma membrane (Fig. 2), but cytoplasmic staining was also observed in a relatively large proportion of the cores.

### Survival

**Fig. 3:**
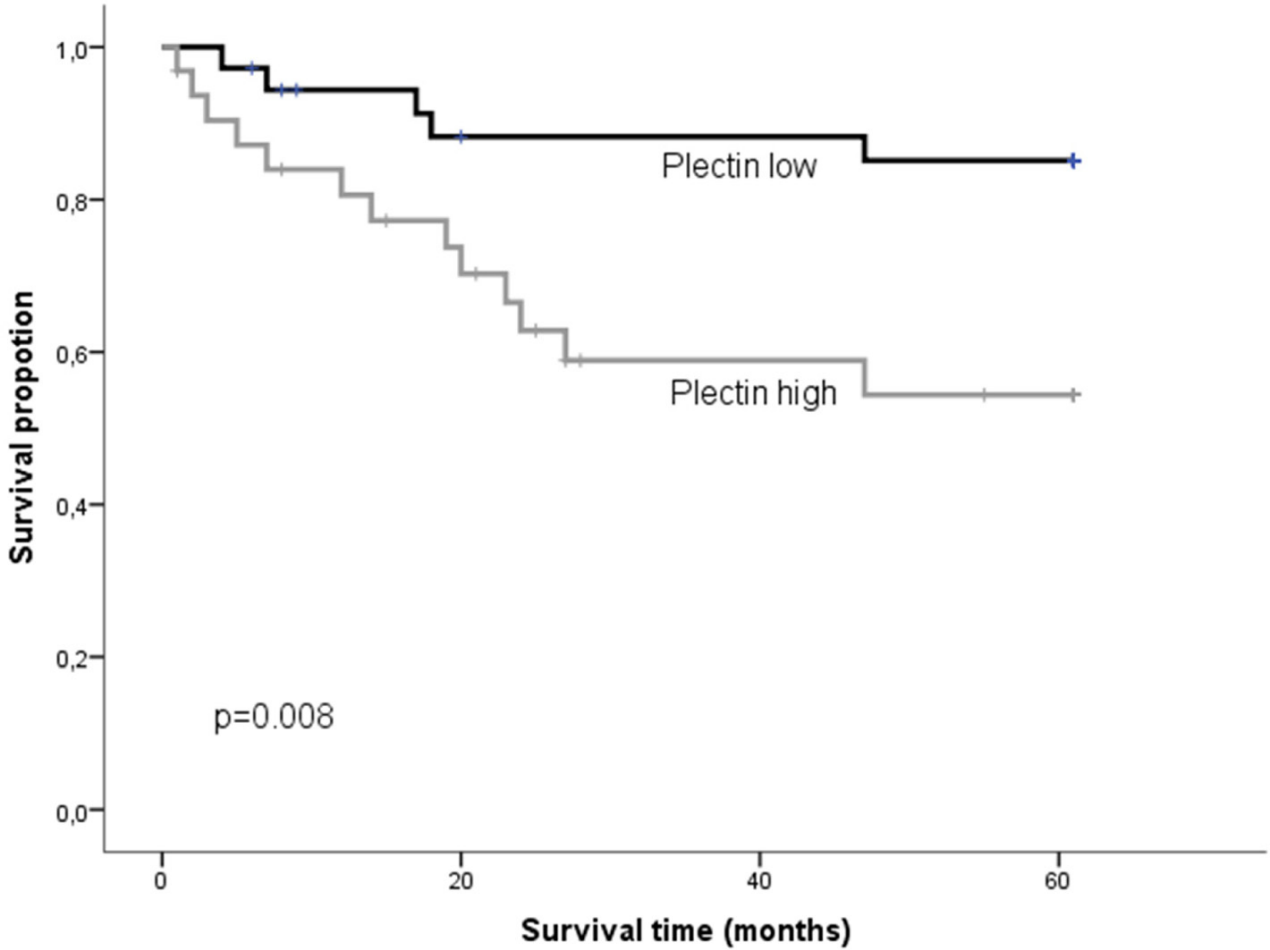
Kaplan-Meier plot. The figure illustrates the difference in survival among N0-patients with high and low expression of plectin respectively

For patients without lymph node metastasis (N0), at any T-stage, the risk of dying of the disease within 5 years was significantly decreased in patients with low plectin expressing tumors compared to those with high plectin expression (*p* = 0.008) (Table 1 and Fig 3). Also, for the patients with T1-tumors, independent of N-status, the statistical analyses revealed a significantly reduced risk for DSD in those who had tumors with low plectin expression (*p* = 0.031). As expected, given the preceding results, patients with T1N0 tumors with low plectin expression had an excellent outcome as all these patients were still alive after 5 years (*p* < 0.001). In a multivariate analysis of the N0-cases including plectin and tumor size (T1 vs T2-4), only high plectin expression was a significant independent predictor of increased DSD (*p* = 0.014, HR 3.674, CI 95 % 1.305-10.344).

**Table 1:** Expression of plectin in the whole cohort, the N0, T1 and T1N0 patients at time of diagnosis. The number of patients with a 5 year disease specific death (DSD) is given in numbers and also as percentage of total in each group of total in each group

		Plectin		
		Low expression	High expression	DSDp
All patients^a^	N (% of total)	55	56	
	5-year DSD	19 (35 %)	25 (45 %)	0.198
N0-cases^b^	N (% of total)	36	32	
	5-year DSD	5 (14 %)	13 (41 %)	0.008*
T1-cases^c^	N (% of total)	23	16	
	5-year DSD	2 (9 %)	6 (38 %)	0.031*
T1N0-cases^d^	N (% of total)	19	9	
	5-year DSD	0	4 (44 %)	<0.001*

^a^total number of patients included in the analysis was 111

^b^total number of patients included in the analysis was 68

^c^total number of patients included in the analysis was 39

^d^total number of patients included in the analysis was 28

*p < 0.05 was regarded as statistically significant, and highlighted in boldsface when present

### Correlation of plectin and uPAR expression

**Fig. 4:**
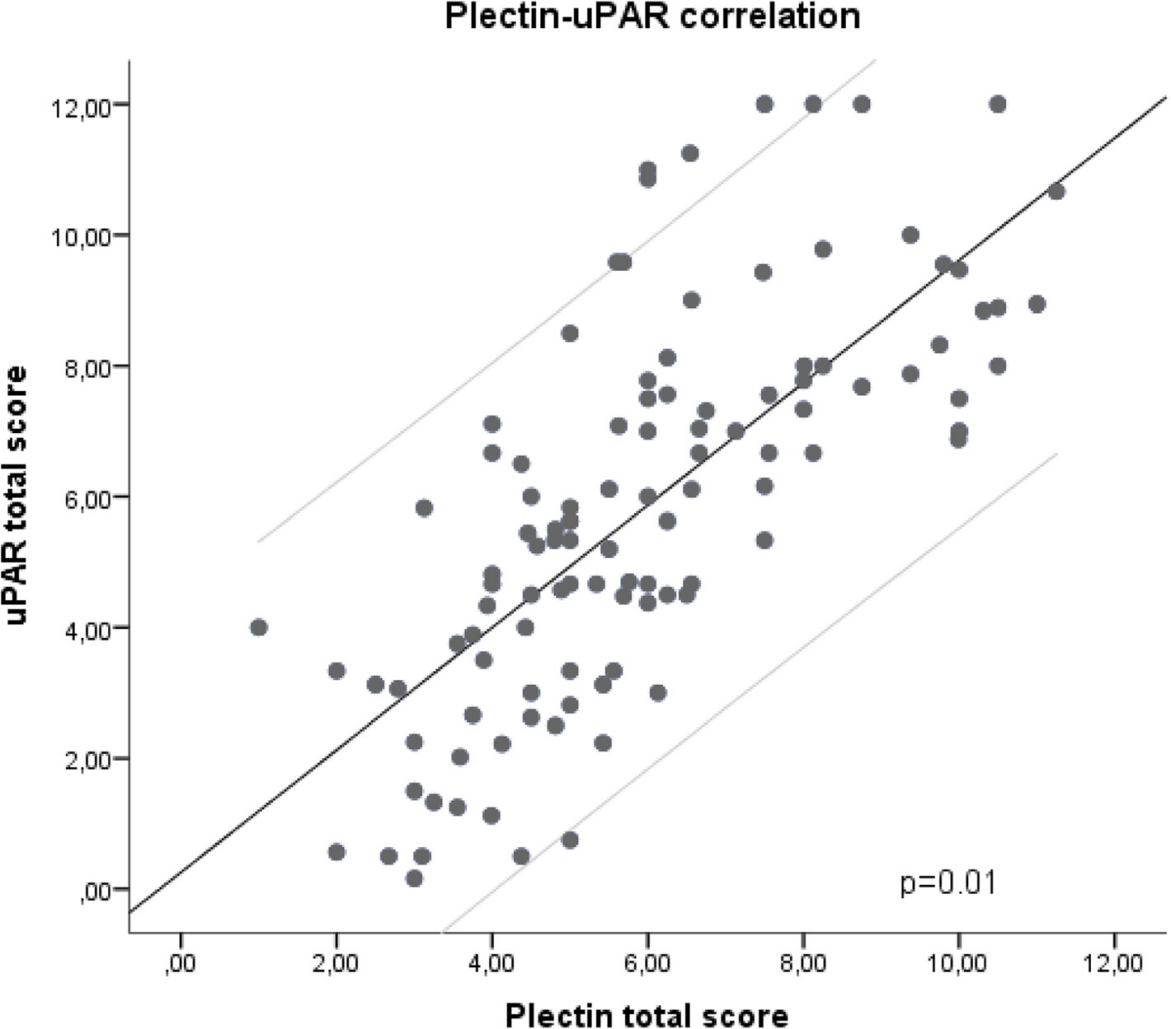
Scatter plot of uPAR and plectin score for the whole cohort. There was a significant correlation (*p* = 0.01) between the plectin and the uPAR score. The light grey lines in the figure represent the 95 % confidence interval

**Fig. 5:**
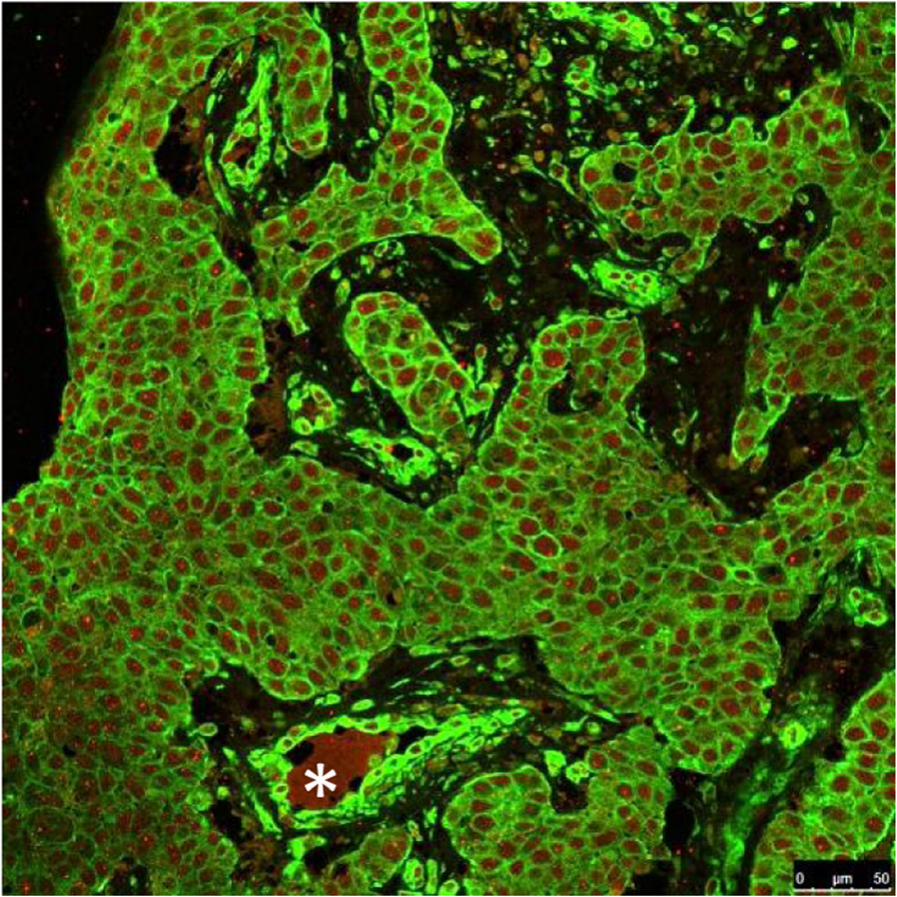
Immunofluorescence staining of oral squamous cell carcinoma tissue. The majority of the cells were positive for both plectin and uPAR. Plectin (green) is located mainly at the plasma membrane and the periphery of the cell, while uPAR (red) is more prominent in the cytoplasmic part of the cells. Plectin is highly expressed in the wall of blood vessels (*asterix*)

We have recently shown that patients with T1N0 tumors expressing low levels of uPAR have a significantly reduced DSD compared to those with a higher uPAR expression. To investigate the distribution of expression of uPAR and plectin in the tumors we did a chi-square test, and found that a significant number of the tumors that were high expressers of uPAR also were high expressers of plectin, and those with low expression of uPAR generally were low expressers of plectin. This correlation was significant with a correlation coefficient of 0.769 (*p* = 0.01) as shown in a scatter plot in Fig. 4. Double immunofluorescence staining was performed on some selected tumors to investigate whether plectin and uPAR were co-located within the same cells, co-expressed by the same cells, or located to the same tumor regions. The immunofluorescence showed that the plectin and uPAR staining was localized to the same areas of the tumor (Fig. 5). As expected, the immunofluorescence staining confirmed that plectin was found mainly at the plasma membrane while uPAR was mainly localized in the cytoplasm.

## Discussion

Surgery is the primary treatment for cancer in the oral cavity, and is often combined with radiotherapy while chemotherapy is more often applied as palliative treatment in late phase of the treatment [35–37]. Side effects of the treatment for oral cancer may be devastating for the patient’s quality of life, [38], and therefore it is important to avoid harmful overtreatment with radiotherapy of patients with small tumors with favorable prognosis. Decisions concerning the extent of treatment of patients with early stage OSCC are often difficult. Still, the choice of treatment is mainly based on TNM-stage which does not discriminate between aggressive and more indolent tumors. Several studies on potential biomarkers for treatment stratification have shown promising results, but so far no such biomarkers have been implemented in clinical practice. In the present study we have investigated the potential role of plectin as a prognostic biomarker. To our knowledge, the prognostic value of plectin in OSCCs has previously only been studied by Katada et al. who investigated a cohort of 62 HNSCC, among them 23 from the oral cavity [22]. Although the cohort was small and heterogeneous, they found that the survival was significantly decreased when the tumor cells expressed high levels of plectin. Our cohort is larger and composed only of tumors from the oral cavity and therefore more homogeneous. In accordance with the results from Katada et al., we found that low expression of plectin in the tumor cells predicts a favorable outcome, but only in patients with early stage disease.

We have previously found that low expression of uPAR correlated with reduced DSD for OSCC patients with T1N0 tumors, and that uPAR therefore might be a suitable prognostic marker for this subgroup of patients [23]. In the present study we found a highly significant correlation between the expression of plectin and uPAR. However, in contrast to uPAR, high expression of plectin correlated significantly with DSD in all patients with non-metastatic disease, and not only the T1N0 subgroup.

Most of the tumor cells that expressed plectin also expressed uPAR. It has previously been suggested that increased expression of uPAR in tumor cells is associated with EMT [27, 29, 39]. Little is known about plectin and EMT, but the formation of plectin-containing podosomes has been proposed as a first step towards EMT in OSCC [40].

## Conclusion

The present study has shown that low expression of plectin predict a favorable outcome for patients with non-metastatic disease. Furthermore, all patients with T1N0 disease and low expression of plectin, survived for more than 5 years. Although the results must be validated in larger cohorts, they suggest plectin as a promising prognostic biomarker, which may be used to guide the treatment stratification for patients with non-metastatic OSCC.

## Abbreviations

DSD: Disease specific death
EMT: Epithelial-mesenchymal transition.
HNSCC: Head and neck squamous carcinoma
OSCC: Oral squamous cell carcinoma
TMA: Tissue microarray
TNM: Classification system to evaluate the extent of primary tumor regional lymph node metastasis and distant metastasis
uPAR: urokinase plasminogen activator receptor
